# Supplementary figures and images for: Identification of the Porcine Vascular Endothelial Cell-Specific Promoter ESAM1.0 Using Transcriptome Analysis
Source: Genes (Basel). 2023 Oct 11;14(10):1928. doi: 10.3390/genes14101928 (PMC10606829; doi:10.3390/genes14101928)

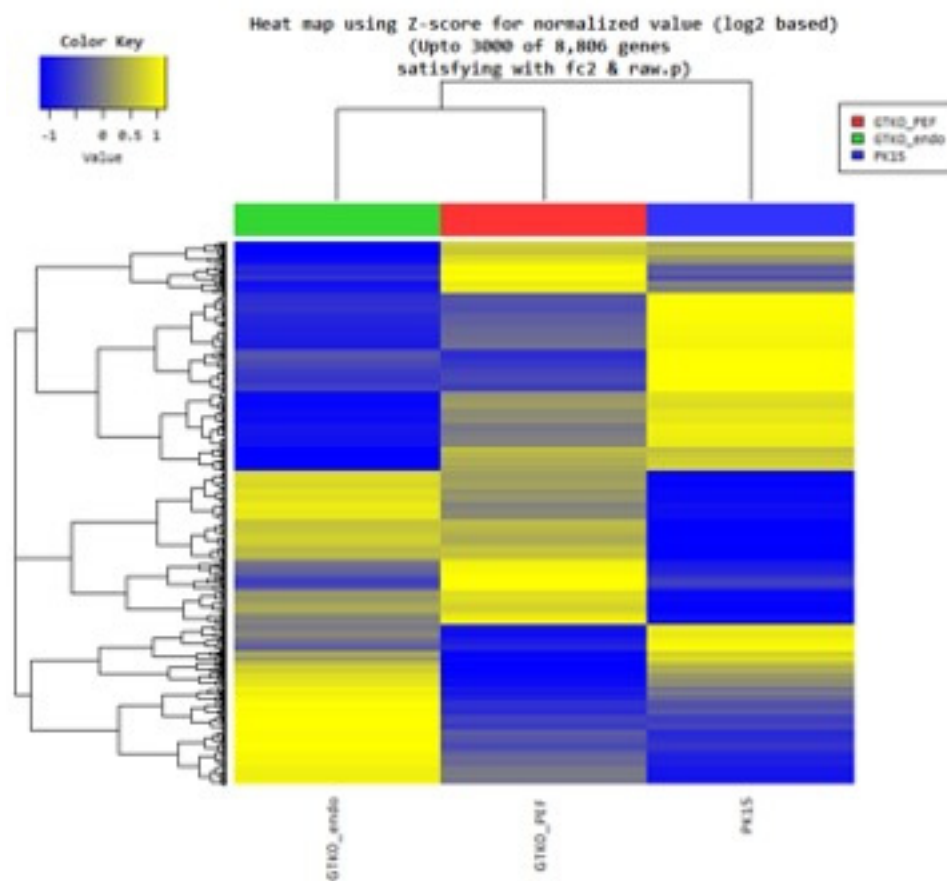

**Figure S1.** Heat map of differentially expressed genes (DEGs).

Supplement: Supplementary file 1 [file genes-14-01928-s001.zip › genes-2623798-Supplementary.pdf]
